# Supplementary figures and images for: Use peripheral blood leukocyte parameters combined with inflammatory indicators in diagnosis and severity assessment of mycoplasma pneumoniae pneumonia in children
Source: PLoS One. 2025 Jun 3;20(6):e0321454. doi: 10.1371/journal.pone.0321454 (PMC12132943; doi:10.1371/journal.pone.0321454)

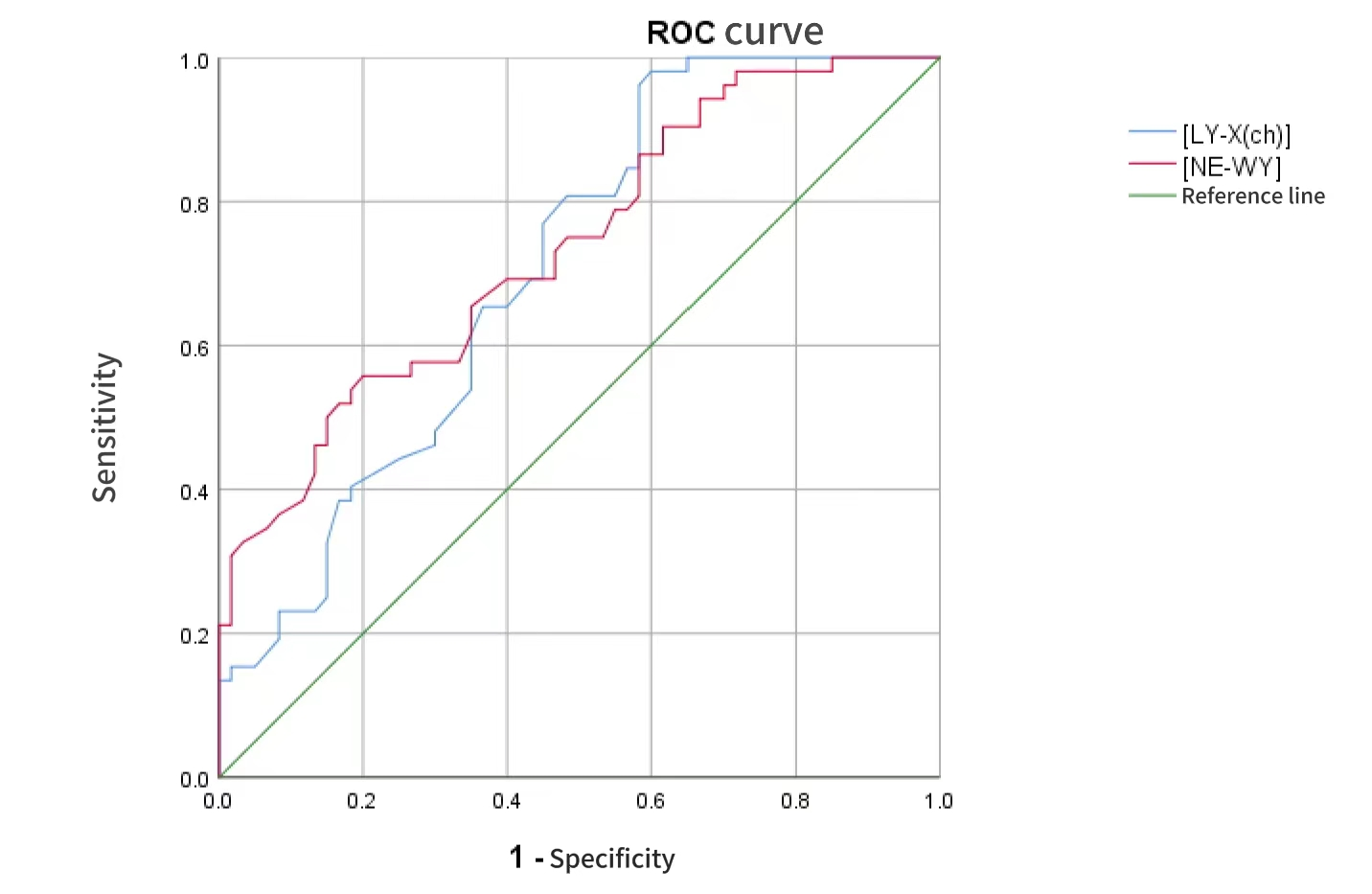

Supplement: S4 Supplementary Figures — (ZIP) [file pone.0321454.s004.zip › Figure1.tif]

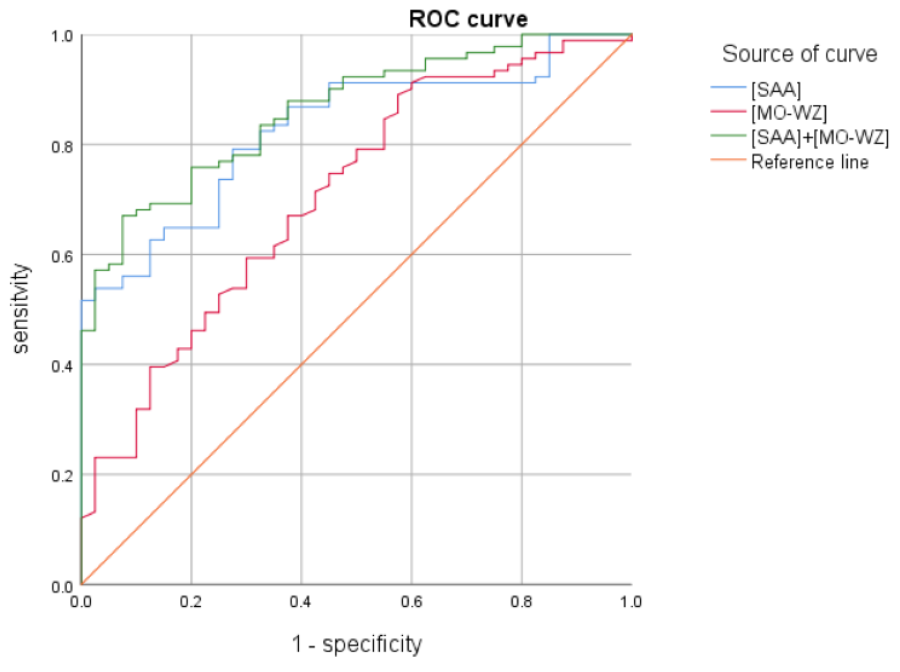

Supplement: S4 Supplementary Figures — (ZIP) [file pone.0321454.s004.zip › Figure2.tif]
