# Supplementary material for: Use peripheral blood leukocyte parameters combined with inflammatory indicators in diagnosis and severity assessment of mycoplasma pneumoniae pneumonia in children
Source: PLoS One. 2025 Jun 3;20(6):e0321454. doi: 10.1371/journal.pone.0321454 (PMC12132943; doi:10.1371/journal.pone.0321454)
Supplement: S5 Supplementary related files — (ZIP) [file pone.0321454.s005.zip › Application form-Chinese.pdf]

临床研究知情同意豁免申请

甘肃省妇幼保健院伦理委员会：

我院王卫凯承担的甘肃省科技重大专项《儿童重症感染早期识别精准诊疗关键技术及推广应用》课题中，有关识别婴幼儿重症感染患者与健康婴幼儿不同检测参数间差异的研究，所涉及的有关数据为婴幼儿感染患者或可疑感染患者及健康体检者正常诊疗过程中需要常规检查所产生的数据及参数，属于回顾性研究范畴，客观上难于全面回顾征求每一位受试者及监护人的知情同意，且豁免受试者及监护人知情不会对受试者权益产生负面影响，特此申请豁免。

研究者承诺如下：

1.任何涉及受试者的信息都将严格保密并进行脱敏处理，不以任何理由提供给与研究无关人员。

2.该研究结果可能会在医学会议上报告以及杂志上发表，但不涉及商业利益，受试者的个人隐私绝对保密，且不包含任何可通过信息获得受试者身份关联的资料。

3.本研究不利用受试者以前已经明确拒绝利用的医疗记录和标本。

基于以上事实情况，请伦理委员会批准该豁免知情同意申请。

主要研究者签字：[illegible]

甘肃省妇幼保健院
检验科
