# Supplementary material for: Use peripheral blood leukocyte parameters combined with inflammatory indicators in diagnosis and severity assessment of mycoplasma pneumoniae pneumonia in children
Source: PLoS One. 2025 Jun 3;20(6):e0321454. doi: 10.1371/journal.pone.0321454 (PMC12132943; doi:10.1371/journal.pone.0321454)
Supplement: S5 Supplementary related files — (ZIP) [file pone.0321454.s005.zip › Application form-English.pdf]

Informed consent waiver

Institutional Review Boards of Gansu Provincial Maternity and Child-care Hospital：

In the project of Key Technologies for Early Identification and Application of accurate Diagnosis and Treatment in Children with Severe Infection undertaken by Wang Weikai of our Hospital in Gansu Province, the research on the difference between different detection parameters of infants with severe infection and healthy infants was carried out. The relevant data involved are the data and parameters generated by infants and young children infected or suspected infected patients and healthy physical examination patients in the normal diagnosis and treatment process, which belongs to the category of retrospective study, and objectively difficult to comprehensively review and solicit the informed consent of every subject and guardian, and the exemption of subjects and guardians from the informed consent will not have a negative impact on the rights and interests of subjects. I hereby apply for an exemption.

The researchers promise the following:

1. Any information involving the subjects will be kept strictly confidential and desensitized, and will not be provided to people not involved in the study for any reason.

2. The results of the study may be reported at medical conferences and published in journals, but it does not involve commercial interests, the personal privacy of the subjects is strictly confidential, and it does not contain any information that can obtain the identity of the subjects through information.

3. This study does not use medical records and specimens that the subjects have previously explicitly refused to use.

Based on the above facts, the Ethics Committee is requested to approve the exemption application for informed consent.

Principal Investigator Signature: [illegible]

甘肃省妇幼保健院
检验科
