# Supplementary material for: Use peripheral blood leukocyte parameters combined with inflammatory indicators in diagnosis and severity assessment of mycoplasma pneumoniae pneumonia in children
Source: PLoS One. 2025 Jun 3;20(6):e0321454. doi: 10.1371/journal.pone.0321454 (PMC12132943; doi:10.1371/journal.pone.0321454)
Supplement: S5 Supplementary related files — (ZIP) [file pone.0321454.s005.zip › Instrument of ratification-Chinese.pdf]

免除知情同意申请审核意见

申请人	张翀
项目名称	儿童重症感染早期识别精准诊疗关键技术及推广应用
项目来源	甘肃省科技重大专项
申请/报告类别	免除知情同意

审核意见：

☑ 经审核确认，本项目符合免除知情同意的条件。

☐ 经审核确认，本项目不符合免除知情同意的条件，请按初始审查申请送审相关材料。

伦理委员会	甘肃省妇幼保健院（甘肃省中心医院）伦理委员会
伦理委员会主任签字	
日期	2024 年 8 月 20 日
