# Supplementary material for: Use peripheral blood leukocyte parameters combined with inflammatory indicators in diagnosis and severity assessment of mycoplasma pneumoniae pneumonia in children
Source: PLoS One. 2025 Jun 3;20(6):e0321454. doi: 10.1371/journal.pone.0321454 (PMC12132943; doi:10.1371/journal.pone.0321454)
Supplement: S5 Supplementary related files — (ZIP) [file pone.0321454.s005.zip › Instrument of ratification-English.pdf]

Exemption from informed consent application review opinion

applicant	zhangchong
Project name	Key technologies for early identification and accurate diagnosis and treatment of severe infection in children and their application
Project source	Major science and technology projects of Gansu Province
Application/reporting categories	Exemption from informed consent

Audit opinion

After audit, it is confirmed that this project meets the conditions of exemption from informed consent.

After audit, it is confirmed that this project does not meet the conditions of exemption from informed consent, please submit relevant materials according to the initial review application

Ethics committee	Institutional Review Boards of Gansu Provi ncial Maternity and Child-care Hospital
Signed by the head of the Ethics committee	[illegible]
Date	August 20, 2024
