# Supplementary material for: Use peripheral blood leukocyte parameters combined with inflammatory indicators in diagnosis and severity assessment of mycoplasma pneumoniae pneumonia in children
Source: PLoS One. 2025 Jun 3;20(6):e0321454. doi: 10.1371/journal.pone.0321454 (PMC12132943; doi:10.1371/journal.pone.0321454)
Supplement: Attachment — Submitted filename: Response to Reviewers 2024.6.13.docx [file pone.0321454.s006.docx]

Dear editor

Thanks for your careful checks, and we will respond to your question:

-Please also ensure that you report in the Methods section the dates when data were accessed for research purposes.

We confirm that the data used for research purposes in the "Methods" section were accessed on January 10, 2024（01.10.2024）

I have resubmitted the manuscript. We appreciate for editors’ careful work earnestly, and hope that the correction will meet with approval. Once again, thank you very much for your comments and suggestions.

Best regards

Mr. Zhang
