# Supplementary material for: Use peripheral blood leukocyte parameters combined with inflammatory indicators in diagnosis and severity assessment of mycoplasma pneumoniae pneumonia in children
Source: PLoS One. 2025 Jun 3;20(6):e0321454. doi: 10.1371/journal.pone.0321454 (PMC12132943; doi:10.1371/journal.pone.0321454)
Supplement: Attachment — Submitted filename: Response to Reviewers.docx [file pone.0321454.s007.docx]

Response Letter

Dear Editor and Reviewer

We sincerely thank the editors and all reviewers for their valuable feedback, which we have used to improve the quality of our manuscript.I would create a table to respond each comment. Sth like the table below:

| Comments | Authors’ reply | Page |
| --- | --- | --- |
| Editor’s comments |  |  |
| *1.Please ensure that your manuscript meets PLOS ONE's style requirements, including those for file naming.* | Our manuscript has been revised in accordance with the stylistic requirements of PLOS ONE, the file name is also changed. |  |
| *2.If you are reporting a retrospective study of medical records or archived samples, please ensure that you have discussed whether all data were fully anonymized before you accessed them and/or whether the IRB or ethics committee waived the requirement for informed consent. If patients provided informed written consent to have data from their medical records used in research, please include this information.* | We have made sure that all data is completely anonymous before we access it, and we have also submitted an informed exemption request to the Ethics Committee, which has been approved, and the specific approval documents have been submitted. |  |
| *3.When you resubmit, please ensure that you provide the correct grant numbers for the awards you received for your study in the ‘Funding Information’ section.* | We have resubmitted the content of the funding information section and made the relevant explanation. |  |
| *4.Please state what role the funders took in the study. If the funders had no role, please state: "The funders had no role in study design, data collection and analysis, decision to publish, or preparation of the manuscript."If this statement is not correct you must amend it as needed. Please include this amended Role of Funder statement in your cover letter; we will change the online submission form on your behalf.* | Statement of the role of funders: The funders had no role in study design,data collection and analysis,decision to publish,or preparation of the manuscript. |  |
| *5.Please remove any funding-related text from the manuscript and let us know how you would like to update your Funding Statement.* | We have removed the content related to funding from the manuscript, and our funding support projects are as follows:Major Project of the Ministry of Science and Technology of Gansu Province,China (grant no.22ZD6FA034) |  |
| *6.Please remove your figures from within your manuscript file, leaving only the individual TIFF/EPS image files, uploaded separately. These will be automatically included in the reviewers’ PDF.* | We have removed the figures from the manuscript and uploaded the figure files separately. |  |
| Reviewer 1’s comments |  |  |
| *1.How was bacterial pneumonia diagnosed in this study, and which bacterial pathogens were included?* | The diagnosis of bacterial pneumonia is made by clinicians combining clinical examination with imaging studies to diagnose pneumonia, followed by multiple PCR pathogen detection to rule out mixed infections of viral and bacterial pneumonia, leading to the diagnosis of bacterial pneumonia[1] Among them, the detection rate of Haemophilus influenae was the highest, 52.5% (27/52), followed by Staphylococcus aureus 27.1% (15/52), Streptococcus pyogenes 9.6% (5/52), Escherichia coli 5.8% (3/52) and Klebsiella pneumoniae 3.8% (2/52). | Page 6, line 9 to page 6, line 17. |
| *2.Mild MPP is self-limiting, and the diagnostic criteria for severe pneumonia also include disease duration factors.* | Regarding the diagnostic criteria for MPP, we reviewed relevant literature[2] The course of mild MPP is generally 7-10 days, while there is no consensus on the course of severe MPP. We found the following about the fever duration in severe MPP: persistent high fever (≥39°C) for ≥5 days or fever for ≥7 days without a decline in peak temperature. | Page 7, line 16 to page 7, line 18. |
| *3.Did the authors consider disease duration factors in the study design, and how did they avoid the influence of disease duration on the study results?* | Regarding the comparison between MPP and bacterial pneumonia, there was no statistically significant difference in the disease duration between the two groups (P>0.05). However, there was a statistically significant difference in the disease duration between the two groups of mild MPP and severe MPP (P<0.05). We did not consider the disease duration of illness because the disease duration is related to the severity of the disease, and the abnormal diagnostic indicators is also related to the severity of the disease. However, the disease duration may not be directly related to the diagnostic indicators. Furthermore, the severity of the disease determines the disease duration. The disease duration is not a factor that leads to the severity of the disease, so we did not consider the disease duration. |  |
| *4.The discussion should explain and discuss the significance of leukocyte parameters.* | We added the significance of leukocyte parameters in the Introduction and Discussion section. | Page 4, line 20 to page 5, line 11 and Page 19, line 6 to page 19, line 19 |
| *5.The manuscript does not provide sufficient information on whether and how the assumptions for each statistical test were checked. This includes assumptions for normality, homogeneity of variances, and independence* | We have supplemented the descriptions related to normality,etc. In this research, statistical approaches were initially employed to determine whether the data conformed to the criteria of normal distribution. The t-test was utilized for quantitative data that adhered to the criteria for normal distribution, whereas the Mann-Whitney U test was applied for non-normal quantitative data. The Pearson's Chi-squared Test was employed for categorical variables. Quantitative data that met the criteria for normal distribution was presented as the mean ± standard deviation (SD), while non-normal distribution data was represented as the median (interquartile range [IQR]). p-value < 0.05 was regarded as statistically significant.  Subsequently, univariate binary Logistic regression analysis was executed, and variables that demonstrated a univariate binary relationship were input into the Multifactorial binary logistic regression, p-value<0.05 was considered statistically significant. Given the number of available events,variables of leukocyte parameters and inflammatory indicators for inclusion were carefully chosen and separately input into the multifactorial binary logistic regression analysis for statistics to avoid overfitting.  Finally, receiver operating characteristic (ROC) curves and the area under the curve (AUC) were plotted to assess the diagnostic efficacy of the white blood cell parameters, p-value <0.05 is considered statistically significant. | Page 9, line 21 to page 10, line 14 |
| *6.The use of multiple logistic regression models without a clear justification for the selection of variables could lead to overfitting, especially given the relatively small sample size in some groups.* |  |  |
| [1] Duan Y., Zhang H., Huang L. The value of traditional methods, fluorescence quantitative PCR, and LAMP in detecting Streptococcus pneumoniae in sputum specimens [J]. Medical Theory and Practice, 2024, 37(16): 2806-2808.  [2] Zhao S., Qian S., Chen Z., et al. Guidelines for the diagnosis and treatment of Mycoplasma pneumoniae pneumonia in children (2023 edition) [J]. Infectious Disease Information, 2023, 36(04): 291-297. | | |

Thank you for your attention.

Yours sincerely

Chong Zhang
