# Supplementary material for: Use peripheral blood leukocyte parameters combined with inflammatory indicators in diagnosis and severity assessment of mycoplasma pneumoniae pneumonia in children
Source: PLoS One. 2025 Jun 3;20(6):e0321454. doi: 10.1371/journal.pone.0321454 (PMC12132943; doi:10.1371/journal.pone.0321454)
Supplement: Attachment — Submitted filename: Response to Reviewers1.25.docx [file pone.0321454.s008.docx]

Response Letter

Dear Editor and Reviewer

We sincerely thank the editors and all reviewers for their valuable feedback, which we have used to improve the quality of our manuscript.I would create a table to respond each comment. Sth like the table below:

| Comments | Authors’ reply | Page |
| --- | --- | --- |
| Editor’s comments |  |  |
| *Please review your reference list to ensure that it is complete and correct. If you have cited papers that have been retracted, please include the rationale for doing so in the manuscript text, or remove these references and replace them with relevant current references. Any changes to the reference list should be mentioned in the rebuttal letter that accompanies your revised manuscript.* | We did not cite the retracted papers. In addition, regarding the first question raised by the reviewer "*A large number of leukocyte parameters (e.g., NE-WY, MO-WX) are presented in the results, but the clinical significance of each is not discussed thoroughly.I suggest the authors focus on the most clinically significant parameters. They should discuss in detail how these specific parameters aid in diagnosing MPP or assessing its severity. For example, they should explain why certain parameters, such as MO-WZ, are more predictive of severe MPP than others.*" We have added other literatures, which have been marked in red in the article. |  |
| Reviewer’s comments |  |  |
| *1. A large number of leukocyte parameters (e.g., NE-WY, MO-WX) are presented in the results, but the clinical significance of each is not discussed thoroughly.I suggest the authors focus on the most clinically significant parameters. They should discuss in detail how these specific parameters aid in diagnosing MPP or assessing its severity. For example, they should explain why certain parameters, such as MO-WZ, are more predictive of severe MPP than others.* | We have discussed the clinical significance of partially supplemented leucocyte parameters (e.g., NE-WY, MO-WX, etc.) and have highlighted them in red. | Page 19, line 5 to page 19, line 16;  Page 22, line 5 to page 22, line 12;  Page 23, line 7 to page 23, line 17. |
| *2.The paper appears to conduct multiple comparisons (e.g., comparisons of various leukocyte parameters and inflammatory markers), but there is no mention of adjustments for multiple testing, such as the Bonferroni correction or False Discovery Rate (FDR) control.I recommend applying correction methods to control the increased risk of Type I errors (false positives) when conducting multiple comparisons. The corrected p-values should be clearly indicated in the table, or the authors should explain the reasons for not making corrections.* | We adjusted the statistical methods as follows:  Kolmogorov-Smirnov test was used to test the normality of each index. ANOVA test was used for normally distributed quantitative data, the Mann-Whitney U test was used for non-normally distributed quantitative data between the two groups, the Kruskal-Wallis test was used for non-normally distributed quantitative data between the multiple groups.After the overall significant difference between multiple groups was compared, Bonferroni correction test was used, and p-value < 0.05 after adjusting the significance was considered to be statistically significant. | Page 9, line 6 -16 |

Thank you for your attention.

Yours sincerely

Chong Zhang
